# Serial Change in Patellar Height after Tension Band Wiring of Patellar Fractures

**DOI:** 10.3390/medicina60050789

**Published:** 2024-05-09

**Authors:** Jin-Ho Cho, Kyung Rae Ko, Seung Jun Park, Sung-Sahn Lee

**Affiliations:** 1 Department of Orthopaedic Surgery, Ilsan Paik Hospital, Inje University School of Medicine, Goyang-si 10380, Republic of Korea; osd07@paik.ac.kr (J.-H.C.); i9914@paik.ac.kr (S.J.P.); 2 Department of Orthopaedic Surgery, Samsung Medical Center, Sungkyunkwan University School of Medicine, Seoul 06351, Republic of Korea; krmd.ko@gmail.com

**Keywords:** patellar fracture, tension band wiring, patellar height, risk factor

## Abstract

*Background and Objectives:* Patella baja is a common complication after operative treatment for patellar fracture. This study aimed to investigate (1) the serial changes in patellar height and (2) the potential predictive factors for patellar height changes after tension band wiring (TBW) for patellar fractures. *Materials and Methods:* Forty-one patients who underwent TBW for patellar fracture between March 2019 and September 2022 were enrolled. To identify serial changes in patellar height, modified Blackburne–Peel index (mBPI) was assessed at just after surgery, at 3 months, at 6 months, at 1 year and at the final follow-up. Multiple regression analysis was conducted to identify factors correlated with mBPI difference between the contralateral side (considered as preoperative status) and injured side. *Results:* The postoperative mBPI exhibited a decline over time (mean mBPI immediately post operation/3 months/6 months/1 year/final follow-up: 0.69/0.63/0.63/0.62/0.61) Specifically, mBPI showed a significant reduction immediately post operation to 3 months (*p* < 0.001), although comparisons at other time points did not reveal significant differences. A lower position of the fracture was associated with a decrease in patellar height after surgery. *Conclusions:* Patellar height was mainly decreased from immediately post operation to 3 months. A fracture in a lower position of associated with decreased patellar height after the TBW of the transverse patellar fracture.

## 1. Introduction

Patellar fractures are a relatively common type of fracture, accounting for approximately 1% of all fractures [1,2]. Their peak incidence occurs during the third to sixth decades of life [3]. Operative treatment is required for about 30% of injured patients, with the primary indication being intra-articular incongruity exceeding 2 mm and loss of the knee joint’s extensor mechanism [4]. As a result, the majority of transverse patellar fractures necessitate surgical intervention. The principal objective in patellar fracture management is the restoration of the knee extensor mechanism. Among the various surgical techniques, tension band wiring (TBW) is generally the preferred method for stabilizing simple transverse fracture patterns. Despite advancements in surgical techniques, clinical and functional outcomes following surgery remain unsatisfactory in a subset of patients [2,5].

Patella baja, also known as patellar infera, is a condition characterized by decreased patellar height. It is composed of a shortened patellar tendon, a decreased distance between the patellar lower pole and the proximal articular surface of the tibia, and a distal position of the patella in the femoral trochlea [6]. Patella baja can occur due to congenital causes or as a result of major knee surgical procedures, femoral nerve injury, or trauma around the knee [7]. The patella lies deeply within the quadriceps and patellar tendon and plays a role in increasing the mechanical advantage of the extensor mechanism [8]. Therefore, alterations in patellar height are associated with changes in the biomechanical extension mechanism. The patella is displaced from the axis of knee joint range of motion and engages with the trochlea at approximately 30 degrees of knee flexion angle. The contact area between the patellar facets and trochlea increases as the knee flexion angle increases. In the case of patella baja, the patella is engaged in the trochlea throughout all ranges of motion rather than disengaging during terminal extension. This alteration could lead to anterior knee pain, impingement, or reduced range of motion [7,8].

Patella baja is a frequent complication following operative treatment for patellar fractures and is associated with limited range of motion in extension and persistent anterior knee pain [9,10]. Previous studies have reported that approximately 12% to 57% of patellar fractures exhibit patella baja after operative treatment [2,11]. The impact of postoperative patellar height on clinical outcomes remains a topic of debate [1,3]. Despite the relatively high incidence of patella baja after surgery, the predictive factors for patella baja have been surprisingly poorly investigated. Furthermore, studies investigating the serial changes in patellar height following TBW surgery have been inadequately demonstrated.

Therefore, the purposes of our study were to investigate (1) the serial changes in patellar height and (2) the potential predictive factors for patellar height change after the TBW of patellar fractures. It was hypothesized that the fracture location was associated with postoperative patellar height.

## 2. Materials and Methods

### 2.1. Study Design and Patients

This retrospective study received approval from our institution’s institutional review board (ISPAIK 2023-10-021). The study encompassed patients who underwent tension band wiring (TBW) for patellar fractures during the period spanning from March 2019 to September 2022. Inclusion criteria comprised patients who (1) underwent TBW for patellar fractures with a displacement exceeding 2 mm and (2) received clinical follow-up exceeding one year. Exclusion criteria included patients who (1) had follow-up periods of less than one year and (2) underwent surgery which applied techniques other than TBW. Out of a total of 56 patients who underwent open reduction and fixation for patellar fractures, 8 patients were subjected to alternative surgical methodologies and 7 patients had follow-up durations of less than one year. Consequently, 41 patients met the eligibility criteria and were enrolled in the current study. The modified Blackburne–Peel index (mBPI) difference between the final follow-up and contralateral measurements was 0.17 (standard deviation: 0.13). When the patients were divided into two groups based on whether the mBPI difference was less than 0.17 (group A) or not (group B); 20 and 21 patients were enrolled in groups A and B, respectively.

### 2.2. Surgical Technique

All surgeries were performed by a single surgeon (S.S.L.) at a single institution. The fractured patella was approached using a midline longitudinal incision. The fracture margins were cleaned, and near-anatomical reduction was performed under fluoroscopic guidance. Two K-wires of 1.6 mm or 1.8 mm were inserted inferosuperiorly and parallel to the long axis of the patella under fluoroscopy. Afterward, an 18 G stainless steel wire was applied to create a figure-of-eight pattern. Additional K-wire fixation was applied for the second fracture fixation in 34C2 pattern fractures following the AO Foundation/Orthopaedic Trauma Association (AO/OTA) system. K-wires were cut and bent and buried in the soft tissue. Blood drainage was not routinely inserted.

Postoperatively, immobilization was enforced for a duration of two days, with controlled range of motion (ROM) allowance of up to 30°, facilitated through the utilization of a hinged knee brace, commencing after the initial two days. Progressive ROM exercises were actively promoted, leading to the attainment of 120° of knee flexion within six weeks. Crutches for partial weight-bearing were permitted for walking during the initial 2 weeks after surgery, and full weight-bearing was permitted at 4 weeks.

### 2.3. Clinical and Radiographic Assessments

Demographic data including age, sex, body mass index, and follow-up period were obtained. Postoperative clinical outcomes were gathered using the following evaluations: ROM, Western Ontario and McMaster Universities Osteoarthritis (WOMAC) index [12,13], and the International Knee Documentation Committee (IKDC) subjective score [14]. Postoperative complications including wire irritation, postoperative infection, non-union, and delayed union were evaluated.

**Figure 1:**
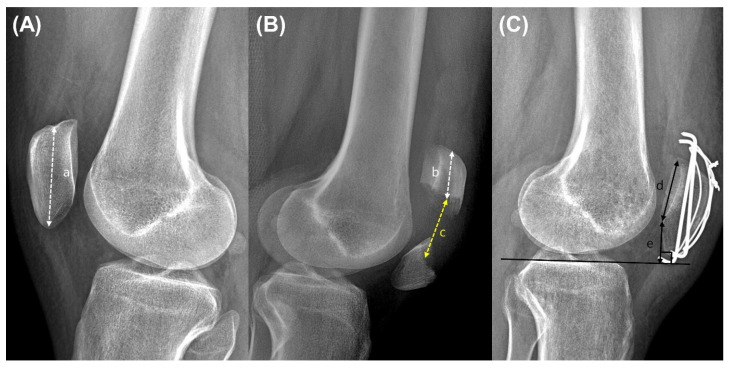
Plain radiographs of (**A**) contralateral side, (**B**) before operation, and (**C**) after operation. Patellar length (a) was measured from the midpoint of patellar upper pole to lower pole at contralateral side radiographs. The upper fragment length (b) was measured from the midpoint of patellar upper pole to lower end of upper fragment. The fracture gap (c) was also measured using lateral radiographs of the injured side before the operation. The fracture gap was defined as the distance between the lowest midpoint of upper fragment and the highest midpoint of lower fragment in lateral plain radiographs. The fracture location was defined as the upper fragment length divided by the patellar length (b divided by a) and expressed as a percentage. The modified Blackburne–Peel index was defined as the perpendicular distance from the lower margin of the patellar articular surface to the tibial plateau line, i.e., (e) divided by the length of the patellar articular surface (d), on lateral-view radiographs.

Preoperative plain radiographs were obtained and analyzed for the classification of fracture pattern following the AO/OTA system and measuring the fracture location and fracture gap. Fracture location was measured using preoperative injured and contralateral side lateral plain radiographs. Patellar length was measured from the midpoint of patellar upper pole to lower pole at contralateral side radiographs. It is difficult to measure preoperative patellar length on the injured side; therefore, patellar length was measured from contralateral side. The upper fragment length was measured from the midpoint of patellar upper pole to lower end of upper fragment at injured side radiographs. Fracture location was defined as the upper fragment length divided by the patellar length and expressed as a percentage. The fracture gap was also measured using injured side radiographs. The fracture gap was defined as the distance between the lowest midpoint of upper fragment and highest midpoint of lower fragment in lateral plain radiographs. To assess patellar height serial change, full extension lateral radiographs—just after surgery and at 3 months, 6 months, 1 year, and final follow-up—were obtained and mBPI was measured. The mBPI [15,16] (normal range: 0.5–0.9) was defined as perpendicular distance from the lower margin of patellar articular surface to the tibial plateau line divided by the length of the patellar articular surface on lateral view radiographs (Figure 1). In studies of the patellar height after planned surgery, like high tibial osteotomy or total knee arthroplasty, preoperative radiographs were usually routinely taken [17]. However, it is difficult to obtain a preoperative radiograph in studies of patellar height after fracture. Therefore, the mBPI of contralateral side was considered the preoperative patellar height. The mBPI difference was defined as the difference between the final follow-up of the injured side and contralateral side measurements to quantify patellar height change between the preoperative measurement and the final follow-up. In the current study, the mean mBPI difference was 0.17. The patients were divided into two groups where the mBPI difference was less than 0.17 (group A) or not (group B). All outcomes were compared between both groups.

All radiographic parameters were measured twice by two orthopedic surgeons, with at least a 4-week interval between each measurement, using a picture archiving and communication system Marosis software (Version 6.0, INFINITT Healthcare, Seoul, Republic of Korea). Intraclass correlation coefficients (ICC) were used to determine the intra- and interobserver reliabilities. All inter- and intra-observer ICCs showed good agreement with respect to the reliability of radiographic measurement (>0.80, Table 1).

### 2.4. Statistical Analysis

Student’s *t*-test for continuous variables and chi-square tests for categorical variables were used to compare the parameters between group A and B. The time-dependent mBPI changes were analyzed using the repeated measures analysis of variance (ANOVA), and post hoc comparisons between the mean extension angles of all pairs of points in time were performed; Bonferroni adjustments were applied to the *p* values to account for multiple testing. Stepwise multiple regression analysis was performed to identify which of the following factors were correlated with mBPI difference: age, sex, body mass index, fracture gap, contralateral mBPI, fracture location, or postoperative ROM. All data were analyzed using SPSS version 27.0 (IBM, Armonk, NY, USA). A *p*-value < 0.05 was considered statistically significant. The primary outcome measure for this study was the difference in mean mBPI during follow-up, calculated with repeated measures ANOVA. It had a power of 99% with an alpha value of 0.05 to detect any significant difference.

## 3. Results

The mean age was 52.4 ± 16.7 years and mean follow-up period was 15.2 ± 4.0 months. Detailed demographic information and fracture classification, in accordance with the AO/OTA system of 2018, are presented in Table 2.

**Figure 2:**
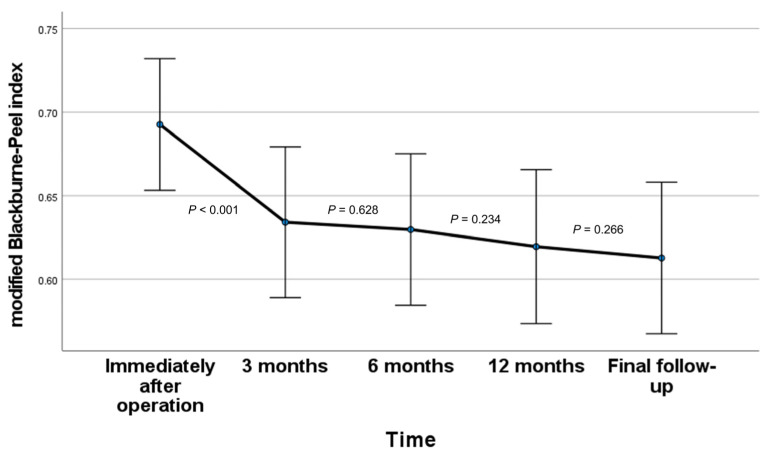
Time-dependent changes in modified Blackburne–Peel index. *p*-values are calculated through post hoc comparisons between two adjacent time points. The postoperative mBPI exhibited a progressive decline over time. A statistically significant reduction in mBPI was observed from immediately after operation to 3 months, while comparisons at other time points did not reveal statistically significant differences.

**Figure 3:**
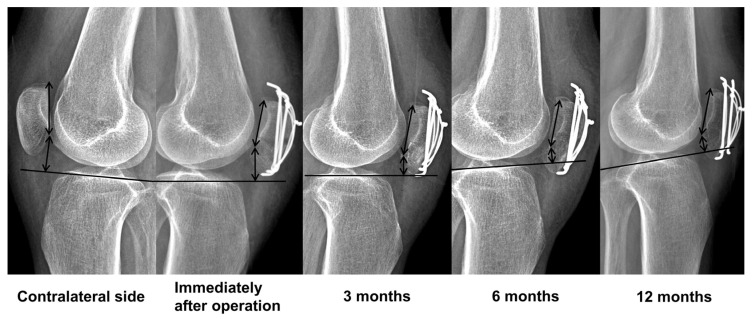
Sixty-year-old female patient injured her patella. The AO/OTA classification was 34C1. The immediate postoperative patellar height was lower than that on the contralateral side. The postoperative mBPI decreased over time.

The contralateral mBPI was 0.78 ± 0.05. Subsequently, the postoperative mBPI exhibited a progressive decline over time (mean mBPI, immediately after operation/3 months/6 months/1 year/final follow-up: 0.69/0.63/0.63/0.62/0.61). Specifically, a statistically significant reduction in mBPI was observed from immediately after operation to 3 months (*p* < 0.001), while comparisons at other time points did not reveal statistically significant differences (Figure 2 and Figure 3).

Age, sex, body mass index, follow-up period, fracture gap, and contralateral mBPI were not significantly different between both groups. Moreover, the patellar height change did not affect the postoperative ROM, IKDC subjective score, and WOMAC index. Interestingly, the fracture location of group B was significantly greater (indicating a lower fracture position) than group A (group A vs. group B: 52.1 ± 16.6 vs. 76.2 ± 10.3; *p* < 0.001, Table 3). In terms of postoperative complications, five cases showed wire irritation. Two and three of the patients were in groups A and B; and there was no statistically significant difference between the groups. There were no other complications.

In multiple regression analysis, to identify predictive factors for mBPI difference, fracture location was the most significant predictive factor for mBPI difference (adjusted *R*^2^ = 0.433, Table 4). In other words, a lower fracture position was associated with decreased patellar height after the TBW of patellar fracture.

## 4. Discussion

A total of 41 patients, whose mean contralateral mBPI was 0.78, were enrolled in the current study. The postoperative mean mBPI immediately after operation/3 months/6 months/1 year/final follow-up were 0.69/0.63/0.63/0.62/0.61, respectively. Among potential risk factors for postoperative patellar height change, fracture location was associated with postoperative patellar baja. The most important finding of our study was that (1) patellar height decreased mainly from immediately after operation to 3 months, and (2) a lower fracture position was associated with decreased patellar height after the TBW of a patellar fracture.

The patella is a triangular-shaped flat and large sesamoid bone situated anterior to the knee joint and presents an anterior surface marked by roughened vertical ridges formed by the quadriceps tendon fibers. Its superior posterior surface is covered with articular hyaline cartilage and can be divided into two facets by a broad, vertical midline ridge. The inferior portion, constituting approximately 25% of the posterior surface, is non-articulating [8]. Functionally, the patella serves as an integral articulating component of the knee joint’s extensor mechanism, transmitting forces from the quadriceps femoris muscle to the patellar tendon, while safeguarding deeper knee joint structures and the quadriceps tendon from frictional forces. Additionally, it provides stability to the knee joint and enhances compressive forces on the extensor mechanism [8,18,19,20]. Biomechanically, the patella acts as a fulcrum, amplifying the lever arm of the quadriceps tendon. This fulcrum action necessitates a pivot surface adapted to withstand high compressive loads with minimal friction [21,22]. Displaced from the axis of knee joint range of motion, the patella becomes the sole component of the extensor mechanism in contact with the femur from full extension to 30 degrees of flexion. Consequently, the effective moment arm of the quadriceps mechanism increases, requiring an additional 60% of torque to achieve the last 15 degrees of knee extension [8,23]. Engagement with the trochlea occurs at approximately 30 degrees of knee flexion, with the contact area between the patellar facets and trochlea expanding as knee flexion increases. At 135 degrees of flexion, the patella enters the intercondylar notch [8,24].

Patella baja, also known as patellar infera, denotes reduced patellar height, characterized by a shortened patellar tendon, decreased distance between the patellar lower pole and the proximal articular surface of the tibia, and a distal positioning of the patella within the femoral trochlea [6]. It manifests in acute and chronic forms, with the acute type often arising postoperatively following knee injuries, surgeries, ligament reconstruction, trauma, arthroplasty, or osteotomy. Chronic patella baja results from quadriceps weakness, immobilization, or reflex sympathetic dystrophy [7,15,16,25,26]. Alterations in patellar height correlate with changes in biomechanical extension mechanisms, wherein normal patellar height disengages the patella from the trochlea during terminal extension, while patella baja maintains engagement throughout all ranges of motion, potentially leading to anterior knee pain, impingement, or restricted motion [7,8].

Mariani et al. investigated the incidence of patella infera after around-knee fracture [11]. They demonstrated that 12% of patellar fractures showed patella infera and suggested that inflammatory or algodystrophic phenomena and quadricep inhibition could be an etiology for postoperatively reduced patellar height. Lazaro et al. reported patella baja in 57% of patients who underwent surgery for patellar fracture [2]. While variations in incidence exist in studies, it is now an established observation that patellar height may diminish following surgical intervention for patellar fractures. The etiology is not fully understood; however, previous studies suggested patella baja resulted from the shortening of the patellar tendon fiber and traumatic and postoperative scarring [9]. Quadricep functional weakness is also one of the factors contributing to the development of patella baja [27]. We think that if transient quadricep functional weakness was the primary cause of patella baja after surgery, patellar height might demonstrate recovery over time. However, our study’s observation that patellar height did not exhibit significant recovery over time indicated that the principal etiology of patella baja following surgery for patellar fractures was likely attributable to patellar tendon shortening due to postoperative scarring, which is consistent with previous suggestions.

It is well known that patella baja can occur after operative treatment for patellar fracture; however, the correlative factors for patella baja have been surprisingly poorly investigated. Sebastian et al. [3] investigated patellar height after patellar fracture with a mean follow-up of 503.8 days. They found 20% and 14.5% of all enrolled patients had patella baja and alta, respectively. The patellar height tends toward low. However, not all patients experienced postoperative patella baja. In our findings, the lower location of the main fracture was associated with inferior postoperative patellar height. As mentioned above, the postoperative scarring and shortening of the patellar tendon were the main reasons for patella baja after surgery for patellar fracture. We think that the increased impact on patellar tendon shortening may be attributable to scarring, which predominantly occurs in lower positions due to the lower location of the fracture. However, because our study was a small-scale study, it is deemed necessary to derive more accurate results through a large-scale study in the future.

The postoperative patients’ clinical or function outcomes were still unsatisfactory in some patients, despite advances in surgical technique [2,5]. Some studies investigated the risk factors for poor postoperative clinical outcomes after the treatment of patellar fractures [3,28]. Vestergaard et al. investigated postoperative patients’ reported outcome measurements (PROMs) using the registry data of Danish Data Protection Agency [28]. They collected postoperative PROMs including the Oxford Knee Score, Forgotten Joint Score-12, and EuroQol 5 Dimensions Quetionnairee—5L. They reported that an age of >40 and female sex were correlated with poor postoperative PROMs after examining 7133 cases of distal femoral, patellar, and proximal tibial fractures. The impact of postoperative patellar height on clinical outcomes or patients’ satisfaction has also been poorly investigated. Sebastian et al. divided patients into three groups, according to postoperative Insall–Salvati ratio, i.e., patella baja, patella alta, and patella norma, after operative treatment for patellar fractures [3]. Of the 54 patients who enrolled their study, 11, 8, and 36 patients were classified into the patella baja, alta, and norma groups, respectively. The three groups were compared using the Munich Knee Questionnaire at last follow-up. There were no significant differences found in the functional outcome according to the Munich Knee Questionnaire. They concluded that different postoperative patella heights did not influence the functional outcomes after surgical treatment for patellar fractures. In our study, groups A and B showed similar WOMAC index scores and IKDC subjective scores, which was concurrent with the previous study. Our findings suggested that postoperative patellar height was not the only factor for postoperative clinical outcomes, and numerous factors could be associated with clinical outcomes. A large-scale study is needed to identify the exact predictive factors for postoperative PROMs after the operative treatment of the transverse patellar fracture. A low preoperative patellar height is associated with postoperative patella infera, which is a risk factor for inferior functional outcomes after total knee arthroplasty or high tibial osteotomy [29,30]. The shortening of the patellar tendon and patellar infera could cause difficulties encountered during later knee surgeries, including arthroplasty and osteotomy, and could be associated with inferior functional outcomes. In our study, patients’ clinical and functional outcomes were not different in terms of patellar height change; however, it could affect functional outcomes after further knee surgeries.

This study has several limitations. First, the patients who enrolled in this study underwent TBW using K-wire and metal wire; therefore, our results cannot be generalized to other surgical or fixation techniques. Further study is needed to identify the influence of different surgical techniques on postoperative patellar height. Second, the mean study follow-up period was 15.2 months, which was relatively short. A long-term study is needed to identify the long-term patellar height change over time. Third, this study was a retrospectively designed study, and seven patients were excluded due to loss of follow-up; therefore, selection or information biases may exist. Fourth, for patellar height evaluation after planned surgery, like high tibial osteotomy or total knee arthroplasty, preoperative radiographs were easily assessed and analyzed. However, it is difficult to obtain a preoperative radiograph in studies of patellar height after fracture. Therefore, the mBPI of the contralateral side was considered the preoperative patellar height in the current study. Since the possibility that the patellar heights on both sides were different before fracture occurred canny be ruled out, this could be a bias in the interpretation of our results. Fifth, our study was a relatively small-scale study. Postoperative clinical outcomes were similar between both groups in our study; however, this might be different in a large-scale study. A large-scale study is needed to identify more exact results. Sixth, measurement errors might have occurred when calculating patellar height because the protocols of the X-rays performed on the same patient at different time points could be slightly different, and the patellar shape could be altered as the fracture healed. Despite these limitations, our study revealed the serial change in patellar height after a patellar fracture and identified the risk factors for postoperative patellar baja after the TBW of a transverse patellar fracture.

## 5. Conclusions

Patellar height mainly decreased from immediately after operation to 3 months after the TBW of a transverse patellar fracture. Of age, sex, body mass index, fracture gap, fracture location, and range of motion, a lower fracture position was associated with decreased patellar height. Postoperative patellar height changes did not influence patients’ clinical outcomes; however, they could affect functional knee outcomes after further knee surgery.

## Figures and Tables

**Table 1:** ICCs of interobserver and intraobserver errors in assessing radiographic measurements (mBPI, modified Blackburne–Peel index).

Measurements	Interobserver	Intraobserver	
		1	2
Fracture gap	0.877	0.882	0.846
Fracture location	0.932	0.911	0.885
Contralateral mBPI	0.861	0.812	0.83
Immediate postoperative mBPI	0.884	0.9	0.875
Postoperative 3 months mBPI	0.843	0.865	0.845
Postoperative 6 months mBPI	0.895	0.837	0.864
Postoperative 12 months mBPI	0.913	0.813	0.892
Final follow-up mBPI	0.88	0.884	0.836

**Table 2:** Demographic data and fracture classification according to AO Foundation/Orthopaedic Trauma Association (AO/OTA) system.

Number of patients	41
Age, year	52.4 ± 16.7 (18–85)
Sex, male/female	22:19
Body mass index, kg/m^2^	23.9 ± 4.0 (18.0–33.8)
Follow-up period, months	15.2 ± 4.0 (11.8–27.1)
Contralateral mBPI	0.78 ± 0.05 (0.66–0.88)
AO/OTA classification	
34A1	1
34C1	26
34C2	14

Continuous variables are presented as mean ± standard deviation (range); mBPI, modified Blackburne–Peel index; AO/OTA, AO Foundation/Orthopaedic Trauma Association.

**Table 3:** Comparison of outcomes between two groups where modified Blackburne–Peel index (mBPI) difference was less than 0.17 (group A) or not (group B).

	Group A mBPI_diff < 0.17	Group B mBPI_diff ≥ 0.17	*p* Value
Number of patients	20	21	
Age, year	47.5 ± 17.5	57.0 ± 14.8	0.069
Sex, male/female	12:8	10:11	0.536
Body mass index, kg/m^2^	24.0 ± 4.6	23.7 ± 3.5	0.824
Follow-up period, months	15.6 ± 4.9	14.8 ± 3.0	0.555
Postoperative ROM, °	136.8 ± 12.2	138.6 ± 6.2	0.547
Postoperative IKDC subjective score	61.8 ± 17.7	56.6 ± 15.7	0.323
Postoperative WOMAC index	11.6 ± 6.6	12.7 ± 5.6	0.573
Fracture gap, mm	17.2 ± 14.4	13.4 ± 8.5	0.323
Contralateral mBPI	0.79 ± 0.06	0.78 ± 0.47	0.245
mBPI difference (contralateral mBPI−final follow-up mBPI)	0.06 ± 0.07	0.30 ± 0.10	<0.001
Fracture location, %	52.1 ± 16.6	76.2 ± 10.3	<0.001
Complications, n	2 (wire irritation)	3 (wire irritation)	0.675

Continuous variables are presented as mean ± standard deviation; WOMAC, Western Ontario and McMaster Universities Osteoarthritis (WOMAC); IKDC, International Knee Documentation Committee; mBPI, modified Blackburne–Peel index.

**Table 4:** The multiple regression analysis of predictive factors affecting the modified Blackburne–Peel index (mBPI) change after tension band wiring for transverse patellar fractures.

Dependent Variable	Independent Variables	Non-Standardized Coefficients		Standardized Coefficients	*p*-Value
		B	SE	B	
mBPI difference	Age	−0.001	0.44		0.398
	Sex	0.025	0.039		0.535
	Body mass index	−0.006	0.005		0.256
	Fracture gap	0.002	0.002		0.344
	Contralateral mBPI	0.368	0.36		0.314
	Fracture location	0.492	0.088	0.669	<0.001
	Postoperative ROM	0.001	0.002		0.438

ROM, range of motion; mBPI, modified Blackburne–Peel index.

## Data Availability

The datasets analyzed during the current study are available from the corresponding author upon reasonable request.

## References

[1] Levack B., Flannagan J.P., Hobbs S. (1985). Results of surgical treatment of patellar fractures. J. Bone Jt. Surg. Br..

[2] Lazaro L.E., Wellman D.S., Sauro G., Pardee N.C., Berkes M.B., Little M.T., Nguyen J.T., Helfet D.L., Lorich D.G. (2013). Outcomes after operative fixation of complete articular patellar fractures: Assessment of functional impairment. J. Bone Jt. Surg. Am..

[3] Sebastian P., Michael Z., Frederik G., Michael M., Marcus W., Moritz C., Peter B., Chlodwig K. (2022). Influence of patella height after patella fracture on clinical outcome: A 13-year period. Arch. Orthop. Trauma. Surg..

[4] Dietz S.O., Hessmann M.H., Gercek E., Rommens P.M. (2009). Patella fracture. Oper. Orthop. Traumatol..

[5] Catalano J.B., Iannacone W.M., Marczyk S., Dalsey R.M., Deutsch L.S., Born C.T., Delong W.G. (1995). Open fractures of the patella: Long-term functional outcome. J. Trauma..

[6] Floren M., Davis J., Peterson M.G., Laskin R.S. (2007). A mini-midvastus capsular approach with patellar displacement decreases the prevalence of patella baja. J. Arthroplast..

[7] Barth K.A., Strickland S.M. (2022). Surgical Treatment of Iatrogenic Patella Baja. Curr. Rev. Musculoskelet. Med..

[8] Fox A.J., Wanivenhaus F., Rodeo S.A. (2012). The basic science of the patella: Structure, composition, and function. J. Knee Surg..

[9] Chang C.H., Chuang H.C., Su W.R., Kuan F.C., Hong C.K., Hsu K.L. (2021). Fracture of the inferior pole of the patella: Tension band wiring versus transosseous reattachment. J. Orthop. Surg. Res..

[10] Kennedy M.I., Aman Z., DePhillipo N.N., LaPrade R.F. (2019). Patellar Tendon Tenotomy for Treatment of Patella Baja and Extension Deficiency. Arthrosc. Tech..

[11] Mariani P.P., Del Signore S., Perugia L. (1994). Early development of patella infera after knee fractures. Knee Surg. Sports Traumatol. Arthrosc..

[12] Clement N.D., Bardgett M., Weir D., Holland J., Gerrand C., Deehan D.J. (2018). What is the Minimum Clinically Important Difference for the WOMAC Index After TKA?. Clin. Orthop. Relat. Res..

[13] Yoon J.R., Yoon T.H., Lee S.H. (2023). The effect of Parkinson’s disease on total knee arthroplasty: A systematic review and meta-analysis. Knee Surg. Relat. Res..

[14] Anderson A.F., Irrgang J.J., Kocher M.S., Mann B.J., Harrast J.J., International Knee Documentation C. (2006). The International Knee Documentation Committee Subjective Knee Evaluation Form: Normative data. Am. J. Sports Med..

[15] Bito H., Takeuchi R., Kumagai K., Aratake M., Saito I., Hayashi R., Sasaki Y., Saito T. (2010). Opening wedge high tibial osteotomy affects both the lateral patellar tilt and patellar height. Knee Surg. Sports Traumatol. Arthrosc..

[16] Seo J.G., Moon Y.W., Kim S.M., Park S.H., Lee B.H., Chang M.J., Jo B.C. (2015). Prevention of pseudo-patella baja during total knee arthroplasty. Knee Surg. Sports Traumatol. Arthrosc..

[17] Lee S.S., So S.Y., Jung E.Y., Kim H.J., Lee B.H., Wang J.H. (2019). Predictive Factors for Patellofemoral Degenerative Progression After Opening-Wedge High Tibial Osteotomy. Arthroscopy.

[18] Freehafer A.A. (1962). A study of the function of the patella. Clin. Orthop..

[19] Kaufer H. (1971). Mechanical function of the patella. J. Bone Jt. Surg. Am..

[20] Grabiner M.D., Koh T.J., Draganich L.F. (1994). Neuromechanics of the patellofemoral joint. Med. Sci. Sports Exerc..

[21] Kwak S.D., Colman W.W., Ateshian G.A., Grelsamer R.P., Henry J.H., Mow V.C. (1997). Anatomy of the human patellofemoral joint articular cartilage: Surface curvature analysis. J. Orthop. Res..

[22] Bellemans J. (2003). Biomechanics of anterior knee pain. Knee.

[23] Lieb F.J., Perry J. (1968). Quadriceps function. An anatomical and mechanical study using amputated limbs. J. Bone Jt. Surg. Am..

[24] Goodfellow J., Hungerford D.S., Zindel M. (1976). Patello-femoral joint mechanics and pathology. 1. Functional anatomy of the patello-femoral joint. J. Bone Jt. Surg. Br..

[25] Nejima S., Kumagai K., Yamada S., Sotozawa M., Inaba Y. (2023). Radiologic simulation of leg length change after double level osteotomy in preoperative surgical planning. Knee Surg. Relat. Res..

[26] Na B.R., Yang H.Y., Seo J.W., Lee C.H., Seon J.K. (2022). Effect of medial open wedge high tibial osteotomy on progression of patellofemoral osteoarthritis. Knee Surg. Relat. Res..

[27] Barbosa R.M., da Silva M.V., Macedo C.S., Santos C.P. (2023). Imaging evaluation of patellofemoral joint instability: A review. Knee Surg. Relat. Res..

[28] Vestergaard V., Schroder H.M., Hare K.B., Toquer P., Troelsen A., Pedersen A.B. (2020). Patient-reported outcomes of 7133 distal femoral, patellar, and proximal tibial fracture patients: A national cross-sectional study with one-, three-, and five-year follow-up. Knee.

[29] Salem K.H., Sheth M.R. (2021). Variables affecting patellar height in patients undergoing primary total knee replacement. Int. Orthop..

[30] Gokay N.S., Erginer R., Dervisoglu S., Yalcin M.B., Gokce A. (2014). Patella infera or patellar tendon adherence after high tibial osteotomy. Knee Surg. Sports Traumatol. Arthrosc..

